# Intensity Inhomogeneity Correction of Structural MR Images: A Data-Driven Approach to Define Input Algorithm Parameters

**DOI:** 10.3389/fninf.2016.00010

**Published:** 2016-03-15

**Authors:** Marco Ganzetti, Nicole Wenderoth, Dante Mantini

**Affiliations:** ^1^Neural Control of Movement Laboratory, ETH ZurichZurich, Switzerland; ^2^Department of Experimental Psychology, University of OxfordOxford, UK; ^3^Laboratory of Movement Control and Neuroplasticity, Katholieke Universiteit LeuvenLeuven, Belgium

**Keywords:** bias correction, bias field, intensity non-uniformity, RF inhomogeneities, magnetic resonance imaging

## Abstract

Intensity non-uniformity (INU) in magnetic resonance (MR) imaging is a major issue when conducting analyses of brain structural properties. An inaccurate INU correction may result in qualitative and quantitative misinterpretations. Several INU correction methods exist, whose performance largely depend on the specific parameter settings that need to be chosen by the user. Here we addressed the question of how to select the best input parameters for a specific INU correction algorithm. Our investigation was based on the INU correction algorithm implemented in SPM, but this can be in principle extended to any other algorithm requiring the selection of input parameters. We conducted a comprehensive comparison of indirect metrics for the assessment of INU correction performance, namely the coefficient of variation of white matter (CV_WM_), the coefficient of variation of gray matter (CV_GM_), and the coefficient of joint variation between white matter and gray matter (CJV). Using simulated MR data, we observed the CJV to be more accurate than CV_WM_ and CV_GM_, provided that the noise level in the INU-corrected image was controlled by means of spatial smoothing. Based on the CJV, we developed a data-driven approach for selecting INU correction parameters, which could effectively work on actual MR images. To this end, we implemented an enhanced procedure for the definition of white and gray matter masks, based on which the CJV was calculated. Our approach was validated using actual T1-weighted images collected with 1.5 T, 3 T, and 7 T MR scanners. We found that our procedure can reliably assist the selection of valid INU correction algorithm parameters, thereby contributing to an enhanced inhomogeneity correction in MR images.

## Introduction

Magnetic Resonance Imaging (MRI) is a technique that delivers detailed images of the human body by analyzing its interactions with radio waves superimposed on a strong magnetic field. Due to high spatial resolution and imaging contrast, MRI has achieved a widespread use in clinical brain imaging. Indeed, it is regularly utilized for the detection of structural changes driven by trauma (Irimia et al., 2012; Sappenfield and Martz, 2013), neurodegenerative disease (Canu et al., 2011; Tillema and Pirko, 2013) and neuropsychiatric disorders (Buchanan et al., 2004; Pomarol-Clotet et al., 2010).

A major drawback in the quantitative as well as qualitative interpretation of structural MR images arises from the presence of artifactual smooth intensity variations across the whole MR image (Belaroussi et al., 2006; Bernstein et al., 2006). These are commonly referred to as intensity non-uniformity (INU), but also *intensity inhomogeneity* or *spatial bias*. According to the radio frequency (RF) field mapping theory, intensity inhomogeneities in MR images can be modeled as multiplicative (Insko and Bolinger, 1993; Stollberger and Wach, 1996). The main factors that can influence the magnitude and spatial profile of the INU include: static field strength, reduced RF coil uniformity, RF penetration, gradient-driven eddy currents, inhomogeneous reception sensitivity profile, and overall subject anatomy and position (Simmons et al., 1994; Mihara et al., 1998; Belaroussi et al., 2006; Vovk et al., 2007). To address this problem, INU correction methods that rely on image features to remove spatial inhomogeneities of different sources have been widely employed by the neuroimaging community (Arnold et al., 2001; Belaroussi et al., 2006; Vovk et al., 2007; Boyes et al., 2008; Zheng et al., 2009; Weiskopf et al., 2011; Uwano et al., 2014).

Since an effective INU correction is critical for investigations of brain structure, previous studies have attempted to compare the performance of several retrospective methods (Velthuizen et al., 1998; Arnold et al., 2001; Likar et al., 2001; Vovk et al., 2006). In the vast majority of studies, INU correction is performed using default parameters. Nonetheless, it is a matter of fact that each method performs better or worse depending on the specific settings used (Boyes et al., 2008; Zheng et al., 2009; Weiskopf et al., 2011; Uwano et al., 2014), and the default configuration may provide in some cases much less accurate results than other ones (Ganzetti et al., 2016). For instance, the definition of optimized parameters is particularly important for the INU correction algorithm implemented in SPM, which is one of the most widely used software for MR data analysis (Ashburner and Friston, 2005). Notably, since the INU correction in SPM is integrated within the brain segmentation tool, an inadequate removal of the INU directly affects the estimate of GM and WM maps (Dawant et al., 1993; Clarke et al., 1995; Pham and Prince, 1999; Zheng et al., 2009). It should be considered that the definition of the best set of parameters for the INU correction algorithm in SPM, as well as for any other alternative INU correction algorithm, is still an unsolved issue.

The optimal set of INU correction parameters can be easily identified on simulated data, for which a direct comparison between true and estimated INU fields is possible. In this case, the correspondence with a ground truth image may be assessed by correlation (Arnold et al., 2001), root mean square error (Ganzetti et al., 2016), L_2_-norm (Chua et al., 2009), and voxel-wise distance (Weiskopf et al., 2011). On the other hand, the use of indirect evaluation metrics, which do not require any reference image, is the only option for actual MR data. Popular indirect measures are based on intensity variability, such as the coefficient of variation of white matter (CV_WM_), the coefficient of variation of gray matter (CV_GM_), and the coefficient of joint variation between white matter and gray matter (CJV). A common premise about the spatial intensity distribution in MR images is that the gray scale distribution of white matter (WM) and gray matter (GM) is somehow defined. Hence, an effective INU correction should theoretically restore the original intensity distribution amplitude, which was altered by the inhomogeneity field. The distribution variability within WM and GM tissues can be separately quantified by CV_WM_ and CV_GM_, respectively. CJV does not only quantify the intensity variability in both WM and GM but also accounts for the overlap between their distributions.

In this study, we evaluate to what extent and how indirect metrics can assist the selection of optimal input parameters for a given INU correction algorithm. We conduct our investigation using the INU correction algorithm implemented in SPM12 (Wellcome Trust Centre for Neuroimaging, University College London), the results of which are particularly sensitive to the selected input parameters (Ganzetti et al., 2016). We focus on T1-weighted images, which are the most commonly used images to investigate brain structure, and the ones typically affected by the INU. We generate simulated MR images with INU fields at different magnitudes and with different image noise levels to define a suitable approach for the detection of algorithm input parameters. Therefore, using the same simulated data, we evaluate the relation between direct and indirect metrics in terms of image quality. After defining an optimized strategy to define INU correction parameters based on an indirect metric, we validate it using actual MR images with different INU spatial profiles and magnitudes.

## Materials and Methods

### Description of the Data-Driven Approach

**FIGURE 1:**
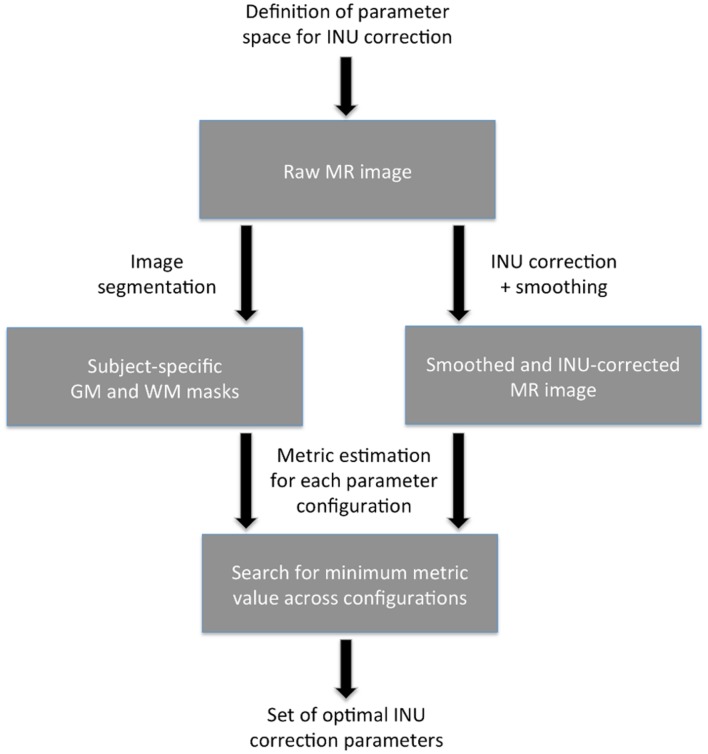
**Flowchart of the data-driven approach**. The set of optimal intensity non-uniformity (INU) correction parameters is identified by searching the minimum performance metric value (either CV_WM_, CV_GM_, or CJV) across values obtained for each combination of the parameters under investigation. The metric value is calculated using subject-specific GM and WM masks and the smoothed INU-corrected MR image.

Our data-driven approach to define optimal parameters for INU correction (see **Figure 1**) requires a raw (unprocessed) structural MR image as input. After defining the whole set of possible INU correction parameters to be examined (parameter space), INU correction and image segmentation are run for each combination of parameters. For each of these runs, the INU-corrected image is spatially smoothed to mitigate the negative effects of noise. In parallel to this, all the gray matter (GM) and white matter (WM) images produced by the image segmentation are processed to derive optimized subject-specific GM and WM masks. After selecting a metric among CV_WM_, CV_GM_ and CJV, the INU correction performance is estimated for each combination of input parameters on the basis of the smoothed and INU-corrected MR image and the subject-specific GM and WM masks. A search for the minimum metric value is conducted, leading to the selection of the set of INU correction parameters putatively yielding the best performance. The software implementing this data-driven approach described above is freely available, and can be found at http://www.bindgroup.eu/index.php/software.

#### Definition of the INU Correction Parameter Space

The INU correction parameters depend on the specific INU correction algorithm chosen. In this study, we tested our approach with the INU correction method implemented in SPM12^1^. This is incorporated within the unified segmentation module (Ashburner and Friston, 2005) and integrated within the ‘Segmentation’ toolbox. The INU correction algorithm is based on two parameters: the *regularization* and the *bias field smoothing*. By decreasing/increasing the *regularization*, the method may be more/less sensitive to sharp intensity transitions between image structures, whereas the *bias field smoothing* permits to model the smoothness of the INU field. For our investigations, we run INU correction on the same image using *regularization* values (0, 10^-5^, 10^-4^, 10^-3^, 10^-2^, 10^-1^, 1, 10) and *bias field smoothing* values (between 30 and 150 mm, sampled at 10 mm intervals) that spanned the whole range suggested by the developers. For a description of the INU correction algorithm and a detailed analysis of its performance, please refer to Ganzetti et al. (2016).

#### Selection of the Indirect Metric

Our approach requires the selection of a metric among CV_WM_, CV_GM_, and CJV to indirectly estimate INU correction performance. These three metrics measure different properties of the image histogram, and are widely used to evaluate to what extent intensity inhomogeneities affect the MR image. They are defined as follows:

(1) $${CV}_{WM}=\frac{\sigma (WM)}{\mu (WM)},{CV}_{GM}=\frac{\sigma (GM)}{\mu (GM)},CJV=\frac{\sigma (WM)+\sigma (GM)}{\mu (WM)-\mu (GM)}$$

where σ and μ indicate the standard deviation and the mean intensity of a given tissue class, respectively. It is commonly accepted that relatively low values of these metrics correspond to smaller presence of INU field and hence better correction performance (Chua et al., 2009).

#### Definition of Optimal Smoothing Level

A drawback concerning the use of CV_WM_, CV_GM_, and CJV is that their values are sensitive to image noise (Chua et al., 2009). Accordingly, the presence of noise in actual MR data limits their reliability when evaluating the INU correction effectiveness. To address this problem, we integrated in our approach spatial smoothing on the INU-corrected MR image. We used the smoothing algorithm implemented in SPM12, and we set the Gaussian smoothing kernel to have full-width at half maximum (FWHM) equal to or smaller than 3 mm in order to avoid excessive image blurring and limit partial volume effects.

#### Definition of Image-Specific GM and WM Masks

A key aspect that hampers an effective use of CV_WM_, CV_GM_, and CJV for real MR data is the fact that optimized masks for WM and GM are not accessible, and that those generated from population-specific templates may not be sufficiently accurate to ensure reliability. Hence, we developed a procedure to address also this problem. For each parameter configuration, the WM and GM probability maps produced by the ‘Segmentation’ toolbox were registered to the SPM template in MNI space, using the deformation field generated by the toolbox itself. Afterward, we binarized the WM and GM probability maps registered to MNI space using a threshold equal to 0.9 to minimize the contaminating effect of partial volume voxels. For each parameter configuration, we calculated the Dice Similarity Index (DSI) between the registered and the SPM template masks for both WM and GM (Zou et al., 2004). The mean DSI (mDSI) for each parameter configuration was computed by averaging the two DSI values for WM and GM, respectively. After estimating the mDSI for each parameter configuration, we selected a relative amount of configurations (called R_T_ hereinafter) that were characterized by the highest mDSIs. For both WM and GM, the probability maps belonging to the selected configurations were averaged together, and the average probability map was thresholded at 0.9 to generate a representative mask. We examined the mDSI of the representative WM and GM masks, obtained for R_T_ ranging from 50 to 100% at intervals of 5%. Thus, using the simulated data, we identified the *R*_T_ value yielding the maximum mDSI value, and consistently used it in subsequent analyses on actual MR data. As such, representative WM and GM mask obtained with the identified *R*_T_ value were considered optimized masks, and employed for the calculation of indirect metrics.

#### Identification of the Optimal Set of INU Correction Parameters

Rather than implementing an iterative algorithm for the determination of the optimal set of INU correction parameters, we opted for a search across the whole space of possible combinations. In first instance, this choice can be justified by the limited problem size, but also by the simplicity of implementation. A number of INU correction algorithms, for instance SPM, typically show relatively similar performance between parameters configurations that are close in the parameter space. These algorithms are therefore suited for the implementation of an iterative search algorithm, which tries to identify a gradient that leads to the configuration with minimum metric value. Nonetheless, there are algorithms, as for example the one implemented in BrainVoyager^2^, for which parameter configurations that are close in the parameter space may have very different accuracy (Ganzetti et al., 2016). The implementation of a search across the whole space of possible combinations may permit to effectively use our data-driven approach with any INU correction algorithm.

### Performance Analysis

#### Testing on Simulated MR Data

##### Creation of simulated MR images

**FIGURE 2:**
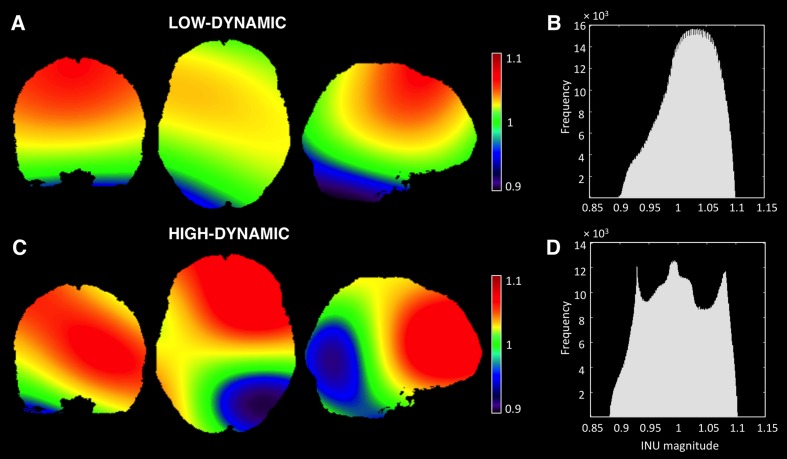
**Simulated INU fields**. Spatial profiles and histograms of the low-dynamic **(A,B)** and the high-dynamic INU fields **(C,D)** at 20% level are represented. Both INU fields are displayed in coronal (*y* = 1), axial (*z* = 0), and sagittal (*x* = 15) sections. It is worth noting that the INU fields at 40 and 80% level are characterized by the same spatial profile of the one at 20%, whereas the field values range from 0.8 to 1.2 and from 0.6 to 1.4, respectively.

Simulated MR data were obtained from the BrainWeb MRI Simulator^3^. First of all, we extracted a realistic INU field map for the T1-w imaging modality, simulated using known spatial varying perturbation of the RF pulse flip angle (Kwan et al., 1999). This map has a smooth spatial profile, reflecting intensity inhomogeneities that are typically observed with lower magnetic field systems, e.g., 1.5 and 3 T MR scanners. The MRI simulator provides an INU field with 20% spatial variation (intensity values between 0.9 and 1.1). For our study, we also generated INU fields with 40 and 80% variation by rescaling the INU profile from the simulator to have values ranging between 0.8 and 1.2 and between 0.6 and 1.4, respectively (**Figure 2A,B**).

In order to generalize our results, we generated an additional intensity inhomogeneity field, characterized by higher dynamics. This profile is intended to mimic better inhomogeneities from higher field scanners. As proposed by Vovk et al. (2004), the field was created by cubic B-spline interpolation between equally spaced nodes at 40 voxels in each direction. Node values, also defined as multiplication factors, were randomly distributed between the same intervals adopted in the previous field (**Figures 2C,D**).

From the BrainWeb MRI simulator we also extracted the phantom volume, which is a simulated MR image representing an anatomical model of a healthy brain. The phantom volume is created by combining ten three-dimensional “fuzzy” tissue membership volumes: GM, WM, cerebrospinal fluid, fat, muscle, skin, skull, glial matter, connective tissue, and background. In each tissue memberships volume, the value of each voxel represents the probability of the tissue to be found at that specific voxel. The MRI simulator combines the tissue membership volumes using weights estimated by Bloch equations (Kwan et al., 1999). These weights are assigned by the simulator depending on the pulse sequence parameters chosen, and can reproduce MR image contrast in a realistic manner (Collins et al., 1998; Kwan et al., 1999). We used default settings of simulator parameters to generate an INU- and noise-free T1-weighted image in order to make our results comparable with previous studies on INU correction (Sled et al., 1998; Arnold et al., 2001; Ashburner and Friston, 2005; Vovk et al., 2005, 2006; Ying et al., 2009; Tustison et al., 2010; Ganzetti et al., 2016). The image was obtained using Spoiled Fast Low Angle Shot (SFLASH) pulse sequence, with TR = 18 ms, TE = 10 ms and α = 30°. The image space was 181 mm × 217 mm × 181 mm, with voxel sampling of 1 mm isotropic. After obtaining the INU field and the INU- and noise-free T1-weighted image from the MRI simulator, we multiplied these to generate an INU-corrupted T1-weighted image. Finally, we also added Rician-distributed noise to the INU-corrupted image. Noise levels were set at 1, 3, and 5% SD compared to the intensity of the brightest tissue in the unbiased image.

##### Performance analysis on simulated data

First, we evaluated the CV_WM_, CV_GM_, and CJV in the identification of the optimized parameter configuration using simulated data with different INU magnitude and noise level. To this end, we adopted WM and GM probability maps provided by the MRI simulator. Before extracting tissue distributions, we thresholded each map at 0.9, in order to control for partial volume effects (Chua et al., 2009). Afterward, σ and μ were computed for both tissues. Finally, we assessed the performance of CV_WM_, CV_GM_, and CJV, at different levels of noise and INU magnitudes.

The direct performance was quantitatively evaluated on the estimated INU field, rather than on the INU-corrected images. In this way, we examined the INU correction results without our performance measures being directly affected by the noise added to the MR images. To account for potential inconsistencies due to arbitrary scaling of the INU estimates, all the INU fields were normalized in intensity (Chua et al., 2009). Normalization was implemented by multiplying the estimated INU field by a scalar value ω, according to the formula by (Chua et al., 2009) as follow:

(2) $$\omega =\frac{\Sigma_{i=1}^{n}\left(b_{sim,i}\cdot b_{est,i}\right)}{\Sigma_{i=1}^{n}{\left(b_{sim,i}\right)}^{2}}$$

where b_sim_, and b_est_ are the simulated and the estimated INU fields, respectively, and n is the number of brain voxels. The deviation (D) of the simulated from the estimated INU fields was then assessed by computing the median of the brain-voxel values in the image *T*, defined as: equation

(3) $$T=\frac{2\left|\omega b_{sim}-b_{est}\right|}{\omega b_{sim}+b_{est}}$$

The smallest *D*-value was associated with the best reconstruction performance (Weiskopf et al., 2011).

To assess the reliability of the information extracted from the indirect metrics, we used two primary indices: (1) the *D*-value obtained for the input parameter configuration providing the lowest metrics value; (2) the Spearman’s correlation coefficient between the matrix of metrics values obtained for all parameter configurations and the corresponding matrix of absolute distances D (matrix-to-matrix correlation, MMC).

#### Testing on Actual MR Images

##### Actual MR images

To validate the proposed approach, we also used T1-w images from three publicly available datasets, acquired at different magnetic field strength in healthy volunteers. The first was the IXI database of the Imperial College London^4^ the second was the KIRBY21 database of the Kirby Research Center for Functional Brain Imaging in Baltimore^5^. This dataset contained images collected in 21 subjects during two different sessions (Landman et al., 2011), which were used in this study for a test–retest analysis. The third dataset, contributed by Dr. Bennett Landman from the Vanderbilt University, was downloaded from the NITRC neuroimaging data repository^6^. MR data belonging to the different datasets were collected in compliance of the requirements set by the review ethical boards of the relevant institutions. Details on scanning parameters for the different datasets are provided in **Table 1**.

**Table 1:** Real data: magnetic resonance (MR) imaging sequence parameters.

	IXI	KIRBY21	NITRC
Scanner	Gyroscan Intera, Philips	Achieva, Philips	Achieva, Philips
Magnetic field (Tesla)	1.5	3	7
Pulse sequence	MPRAGE	MPRAGE	3D TFE
Coil	Standard TMJ coil	8-channel coil	16-channel coil
TR (ms)	9.8	6.7	5.5
TE (ms)	4.6	3.1	2.6
Flip angle (degrees)	8	8	7
Inplane resolution (mm)	0.94 × 0.94	1 × 1	0.7 × 0.7
Slice thickness (mm)	1.2	1.2	0.7

*MR images from different datasets (IXI, KIRBY21, NITRC) were used in this study. The three datasets are characterized by a different static field magnitude. The main imaging parameters of each sequence are reported in the table. TR, repetition time; TE, echo time; α, flip angle; MPRAGE, magnetization-prepared rapid gradient-echo; 3D TFE, Three-dimensional Turbo Field Echo; TMJ, Temporomandibular joint*.

##### Performance analysis on actual data

On actual MR data, we run INU correction and segmentation using the same range of input parameters used with simulated data. First, we computed the segmented WM and GM probability maps for each parameter configuration and generated optimized masks (see the procedure described in Definition of Image-Specific GM and WM Masks). Then, we estimated the relative noise as compared to the signal intensity in each image under investigation. We quantified both signal and noise on an axial slice cutting the corpus callosum at both ends: the signal corresponded to the maximum intensity within a polygonal ROI at the anterior end of the corpus callosum, and the noise to the standard deviation of the intensity within a circular ROI of 10 mm radius located outside the brain. Based on the estimated noise level, we defined the necessary level of smoothing based on the results of our simulations and we applied to the actual MR volumes. Finally, the optimized masks and the spatially smoothed MR images were then used to calculate indirect metric values, searching for the input parameter configuration potentially yielding the most accurate results.

We checked the accuracy of the INU correction results by visual inspection of the INU-corrected T1-weighted images, as well as the reconstructed INU profile. Importantly, we verified that MR images with higher dynamics in the INU profile (typically associated with a MR scanner with higher static field) lead to the definition of smaller regularization values, and possibly smoothing values. Also, we used the whole set of images from KIRBY21 dataset to conduct a test–retest analysis, aimed at examining whether the INU correction with the parameters determined using our data-driven approach leads to increased image reproducibility compared to the default configuration. To this end, we used as a quantitative index the DSI between GM (and WM) masks in MNI space, derived from each of the two sessions. We assessed significant increases/decreases in DSI values between sessions at the group-level by means of paired *t*-tests.

## Results

**FIGURE 3:**
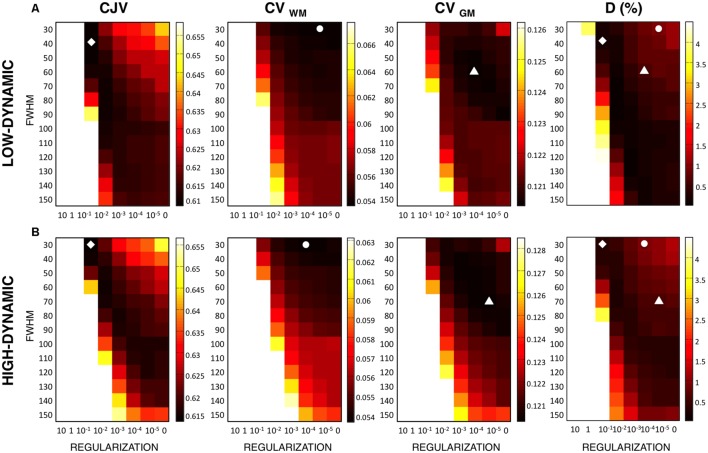
**Metric dependence on the INU correction results produced by different input parameters**. In order to assess the performance of intensity inhomogeneity correction for each indirect metric, we analyzed several parameter configurations. In SPM, the *regularization* and the *bias field smoothing (FWHM)* parameters were varied accordingly. We computed the voxel-wise distance D between the simulated and the estimated INU field for each configuration, which was used as a reference. CJV (indicated with a diamond marker), CV_WM_ (indicated with the circle marker), and CV_GM_ (indicated with the triangle marker) are shown for the low-dynamic **(A)** and high-dynamic **(B)** profiles. The results shown in figure refer to the simulated MR dataset with 40% INU and 1% noise level.

We started our investigations by using the simulated T1-weighted image with INU 40% relative magnitude and 1% noise level, and examining the variability of CJV, CV_WM_, and CV_GM_ across different configurations of input algorithm parameters. For each metric, the configuration with the lowest value (associated with the putatively best INU estimate) was identified and its accuracy was quantified by comparing the corresponding INU against the simulated INU (**Figure 3**). This analysis revealed that the CJV generally provides lower absolute distances, and therefore more accurate results than CV_WM_ and CV_GM_. For CJV, CV_WM_, and CV_GM_ calculated on low-dynamic profile MR images, D was 0.6, 1.4, and 1.1%, respectively. For the high-dynamic profile, it was 0.8, 1.5, and 1.1%, respectively. Not only did a smaller absolute distance characterize the selected parameter configuration, but also the matrix patterns better resemble the matrix of absolute distances D. For the low-dynamic profile, MMC was 0.96, 0.57, and 0.63 for CJV, CV_WM_ and CV_GM_, respectively; for the high-dynamic profile, MMC was 0.99, 0.72, and 0.78 for CJV, CV_WM_, and CV_GM_, respectively.

**FIGURE 4:**
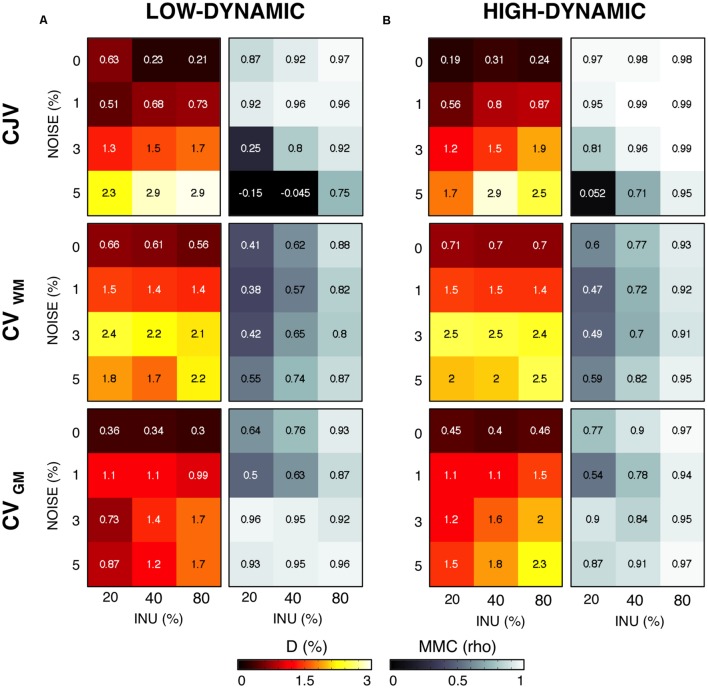
**Sensitivity of different metrics to inhomogeneity magnitude and noise**. For each INU field magnitudes and noise level, we calculated the voxel-wise distance D between the simulated and the estimated INU fields, as well as the matrix-to-matrix correlation (MMC). We report in this figure the results for the parameter configuration identified by each metric. CJV, CV_WM_, and CV_GM_ were compared for the low-dynamic **(A)** and high-dynamic **(B)** INU profiles.

Then, we evaluated the performance of the three metrics for different levels of INU field magnitude and image noise (**Figure 4**). As before, the absolute distance D for the parameter configuration selected from a given metric was computed, as well the MMC between the metric and the distance matrices. An increased INU field magnitude and/or an increase image noise level generally yielded higher D for all the metrics. CJV generally outperformed the others at low noise levels regardless the INU field magnitude and the spatial profile, but was relatively less effective on high-noise MR images. In turn, CV_GM_ showed good stability at higher noise levels. CV_WM_ underperformed the other two metrics for most of the INU and noise levels.

By means of a two-way analysis of variance (ANOVA) we examined the influence of noise and INU magnitude on the absolute distance D. For both INU profiles, the effect of noise was highly significant (*F* = 367.28, *p* < 0.001 for the low dynamic, *F* = 50.6, *p* = 0.001 for the high dynamic), whereas the INU magnitude showed a much less significant effect (*F* = 7.56, *p* = 0.0229 for the low-dynamic profile, *F* = 3.87, *p* = 0.0834 for the high-dynamic one). We did not investigate further the dependence of the metrics on the INU magnitude, and reported from this point on only average performance over INU levels.

**FIGURE 5:**
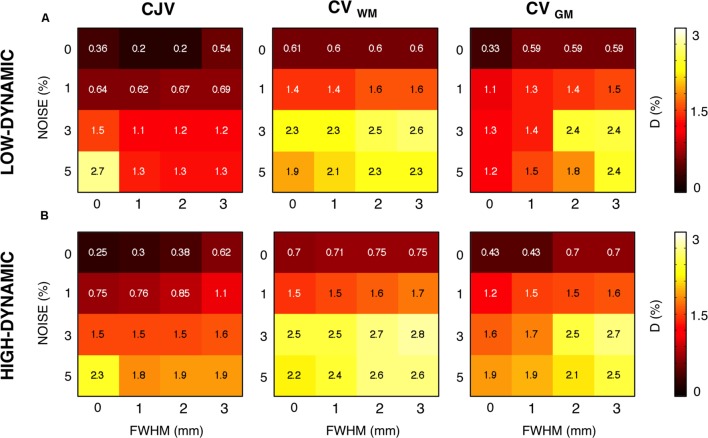
**Relation between spatial smoothing and image noise**. We assessed the relation between image noise and amount of smoothing applied after INU correction. The voxel-wise distance D between the simulated and the estimated INU fields was computed. We report in this figure the results for the parameter configuration identified by each metric. CJV, CV_WM_, and CV_GM_ were compared for the low-dynamic **(A)** and high-dynamic **(B)** INU profiles.

Next, we evaluated to what extent and how spatial smoothing can influence an accurate INU reconstruction (**Figure 5**). For a smoothing level set at 1 mm FWHM, a marked improvement of CJV, and no clear changes of CV_WM_ and CV_GM_ values, were found. Notably, a smoothing larger than 2 mm of FWHM led to less accurate INU reconstructions. This was evident in CV_WM_ and CV_GM_, and less pronounced in CJV measures. Based on these results, we selected the CJV to identify input configurations with low INU estimation errors, and conducted further analyses on CJV only.

**FIGURE 6:**
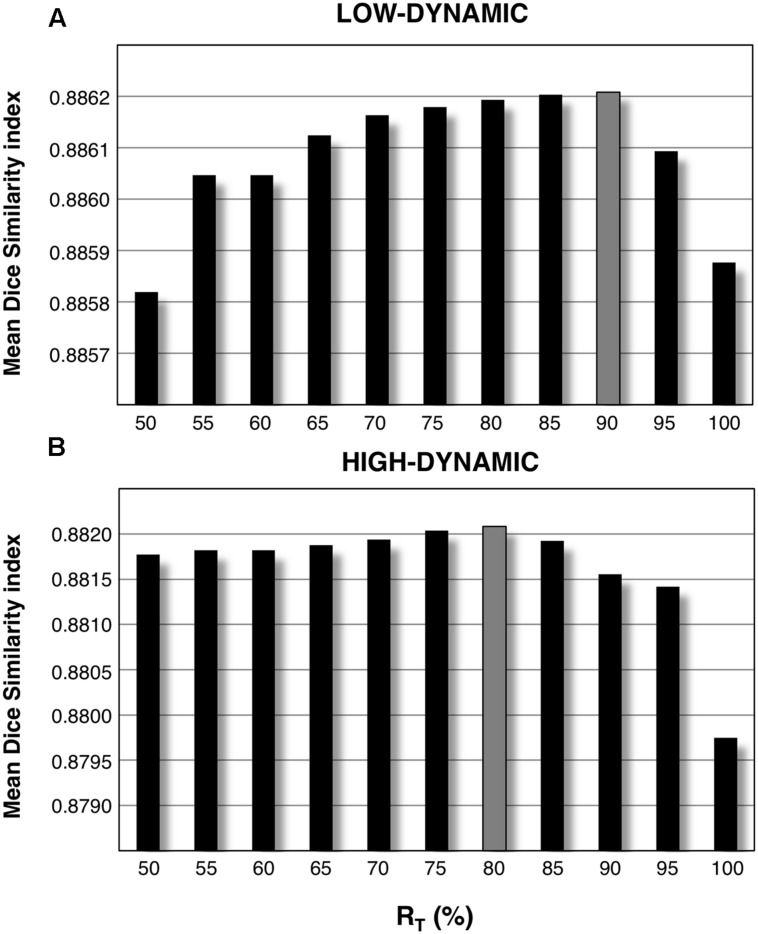
**Mean Dice Similarity Index (mDSI) threshold analysis**. By defining a subset of segmented WM and GM masks corresponding to each parameter configuration and averaging them together, we generated an improved version of the same masks. This was separately done for low-dynamic **(A)** and high-dynamic **(B)** INU profiles, using a relative amount of configurations R_T_ ranging from 50 to 100%. Each of these values represents the relative amount of included masks with respect to the total number of parameter configurations. The bar plots represent the mDSI, which quantifies the correspondence of each segmented mask with respect to the SPM template mask. The mDSI shown in figure was calculated for the two INU field profiles, averaging together the results over the whole set of simulated data (12 simulated images: 3 INU field magnitudes × 4 noise levels).

We addressed the issue of defining subject-based masks to enhance the use of CJV in actual MR images. GM and WM probability maps corresponding to each of the parameter configurations under investigation were estimated, and a subset of them was used to generate average WM and GM masks. Our analysis on both low and high dynamic INU profiles revealed that, on average, including 85% of the masks with the largest correspondence with the SPM template mask in individual space (*R*_T_ equal to 85%) is likely to be a reliable approach to ensure an effective use of the CJV (**Figure 6**).

**FIGURE 7:**
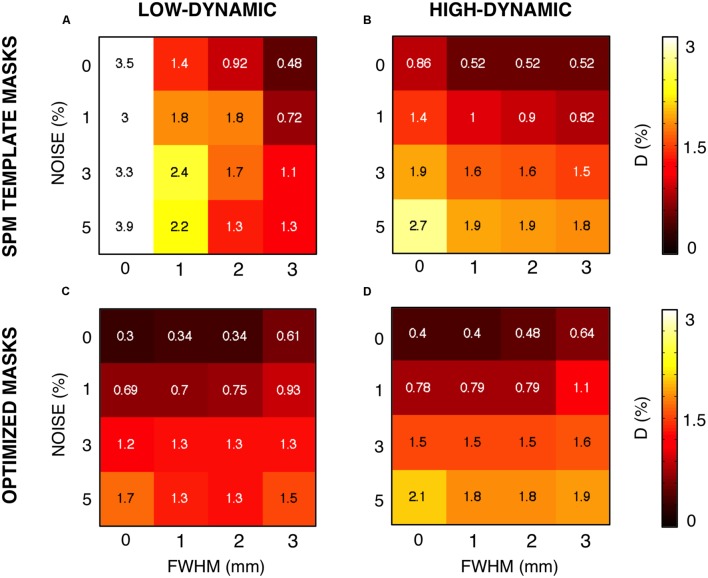
**Coefficient of joint variation (CJV) results obtained using optimized and standard masks**. We assessed the impact of the masks on the CJV results, expressed in terms of the voxel-wise distance D between the simulated and the estimated INU fields. Specifically, we compared the results obtained using the MNI template masks **(A,B)** and the optimized masks **(C,D)**. We examined the performance for different noise level and smoothing, both for low- **(A,C)** and high-dynamic **(B,D)** INU profiles.

The need of a procedure for the definition of reliable WM and GM masks was confirmed by a complementary analysis conducted on the CJV, using the template (not subject-specific) masks derived from SPM (**Figure 7**). When comparing *D*-values obtained using the SPM template masks and the average-based individual masks, the performance obtained using the former was found to be much inferior. On the other hand, by implementing our data-driven procedure, it was possible to achieve performance similar to the ones derived from the BrainWeb simulator masks used in the first part of the study (for comparison, see **Figure 5**).

**FIGURE 8:**
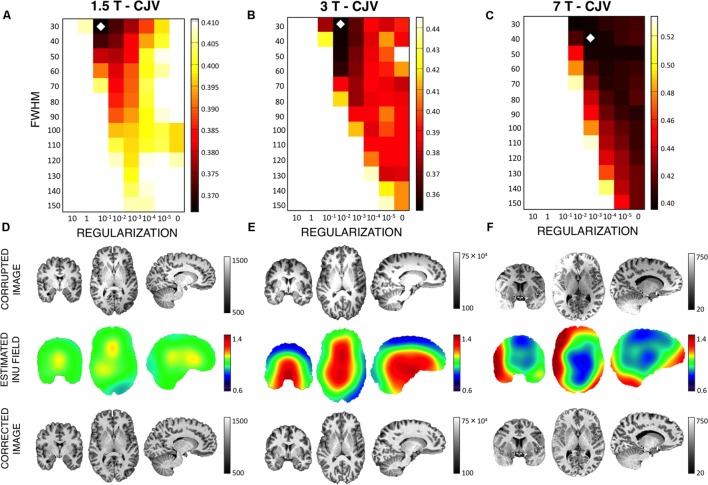
**Intensity non-uniformity correction on actual MR images**. We examined the effectiveness of the CJV analysis on actual MR data, after smoothing, and mask optimizations, for three representative images collected using a 1.5 T, a 3 T, and a 7T MR scanner **(A–C)**, respectively. The diamond marker highlights the input parameter configuration selected on the basis of the CJV results. INU-corrupted image, estimated INU field and INU-corrected image are shown for each dataset **(D–F)**.

**FIGURE 9:**
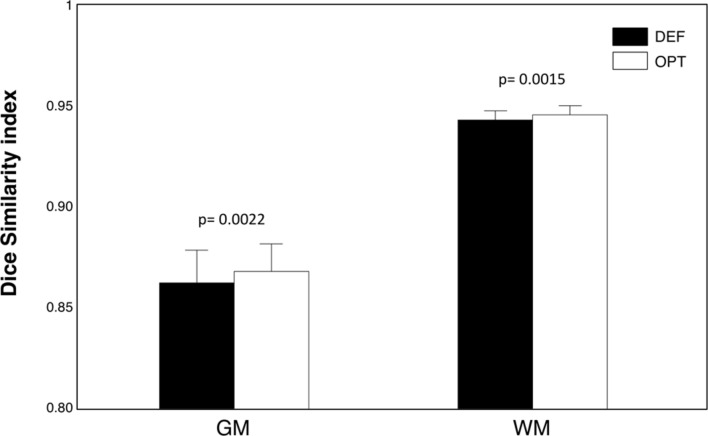
**Test–Retest analysis of INU correction performance**. We used the full set of KIRBY21 images to perform a test–retest reliability analysis. As an indirect measure of INU correction performance, we employed the DSI between GM (and WM) segmented volumes. We compared the DSI values obtained from the optimized and default parameter configurations using paired *t*-tests. The bar plots show mean and standard error for GM and WM masks, and for optimized (OPT) and default (DEF) configurations, respectively. The probabilities estimated using the *t*-tests are indicated in the figure as well.

To validate the usefulness of the data-driven approach for the input parameters definition, three MR images, collected with 1.5 T, 3 T, and 7 T scanners, respectively (**Figure 8**), were used. The noise level was 1.38, 1.25, 0.72% for the three images, respectively. As such, a smoothing level equal to 1 mm FWHM was used to estimate the CJV. Then, WM and GM masks were generated with a relative amount of configurations R_T_ equal to 85%. The analysis of CJV values obtained using different input parameters for the INU correction algorithm revealed different solutions for the three MR images under investigation. The 1.5 T dataset was characterized by a smoothing parameter of 30 mm FWHM and a regularization parameter of 0.1, consistent with the low-dynamic profile. This was supported by a visual inspection of the raw data, which also showed a negligible INU magnitude. A parameter matrix mainly weighted to higher regularization values characterized the 7 T image, which had a highly dynamic spatial profile. In this case, the identified regularization parameter was equal to 0.001. The INU for the 3 T image had intermediate magnitude compared to those of thee 1.5 T and 7 T images, as well as low dynamic profile. The analysis of the CJV suggested the regularization parameter to be best set to 0.01, with a smoothing parameter of 30 mm FWHM. When we extended this analysis of all the 42 MR images collected at 3 T and belonging to the KIRBY21 dataset, our data-driven approach was found to yield the same parameter configuration (smoothing level: 30 mm FWHM; regularization parameter: 0.01). By using the KIRBY21 dataset, we also tested whether our approach yielded increased INU correction reliability. Notably, a significant increase was observed in the test–retest DSI analysis for both GM and WM masks (**Figure 9**) when using the optimized configuration as compared to the default one. Specifically, the average DSI across subjects increased from 0.862 to 0.8675 for GM (*p* = 0.0022) and from 0.9406 to 0.9432 for WM (*p* = 0.0015).

## Discussion

Intensity non-uniformity correction is a fundamental processing step for structural MR images. It is a matter of fact that the performance of any INU correction method depends on the input setting used (Boyes et al., 2008; Zheng et al., 2009; Weiskopf et al., 2011; Uwano et al., 2014), and a less effective INU correction can substantially hamper the reliability of MR imaging results (Pham and Prince, 1999; Ashburner and Friston, 2000; Good et al., 2001; Zheng et al., 2009). Using simulated MR data, we previously showed that the INU correction using specific parameter configurations may be much more accurate than those obtained using a default one (Ganzetti et al., 2016). However, to the best of our knowledge no reliable method exists to define parameters that most likely yield the best correction for actual MR data. Here we examined the characteristics of different metrics, defined among them the most accurate one, and used it to develop a data-driven approach to address this problem. We conducted our investigation using the INU correction algorithm implemented in SPM12, which is one of the most widely used software for MR data analysis. Notably, this algorithm would largely benefit from an optimization approach, as it is largely sensitive to the selection of the input parameters, namely the regularization and the smoothing factor (Ganzetti et al., 2016).

In actual MR data, a common approach to assess INU correction is the one based on indirect measures relying on intensity variability. Among them, the CJV, the CV_WM_ and the CV_GM_ metrics are the most commonly used ones in the literature. Specifically, the CV expresses the normalized standard deviation in a single tissue class, whereas the CJV takes into account the intensity distributions in both classes. In line of principle, the smallest CV and CJV correspond to smaller intensity inhomogeneity residual, thus better performance (Likar et al., 2001; Belaroussi et al., 2006). On the other hand, the CJV not only evaluates the parallel reduction of GM and WM distributions, but also the degree of overlap between the two. Indeed, an effective INU correction produces a consistent increment of contrast in the image, reflected by a clear separation of WM and GM distribution peaks, and thus a decrease of CJV. In addition, a strong INU correction may remove smooth intensity variations characterizing the actual anatomical contrast. In this scenario, while CV_GM_ and CV_WM_ decrease, the CJV increases because the WM and GM distributions peaks get closer. Our simulation results on both voxel-wise distance (D) and matrix-to-matrix correlation (MMC) revealed a larger accuracy of CJV compared to the other two, regardless of the spatial profile of the INU (**Figure 3**). CJV combines information about image intensities in both GM and WM. In this manner, it allows the joint assessment of intensity variability within each tissue class as well as in the image contrast between the two structures. In turn, CV_WM_ and CV_GM_ are estimates derived from image values only in WM and GM, respectively. When the INU correction tends to overestimate the actual inhomogeneities present in the MR image, the contrast diminishes and the CV may erroneously detect an image improvement simply due to a reduced standard deviation in the intensity distribution. This effect may explain - at least in part - the results obtained on simulated data, for which CV_WM_ and CV_GM_ tended to indicate low regularization values and low smoothing factors as yielding better INU correction (**Figure 3**). Specifically, lower values of regularization allow the INU correction algorithm to follow sharp intensity variations, up to the point that factual anatomical variations may be canceled. Our findings suggest that considering the overlap between the intensity distributions of distinct tissue classes is very important for the detection of INU correction performance, and that the CJV may be potentially more suitable than CV_WM_ and CV_GM_ for an accurate inhomogeneity correction.

It should be considered that actual MR images may be characterized by various noise levels and INU magnitudes. These may depend on the subject as well as on the acquisition hardware and sequence used. Our findings suggested noise to substantially influence the performance of CJV, CV_WM_, and CV_GM_ (**Figure 4**). Specifically, all the three metrics provided accurate results for low levels of noise (0–1%), with the CJV overperforming CV_WM_ and CV_GM_ both in terms of D and MMC. On the other hand, the CJV was the most sensitive to noise, and underperformed CV_WM_ and CV_GM_ with very noisy MR data (5% noise level). A possible explanation may be the spreading effect spatial noise has in the intensity distribution of both WM and GM. This may hamper a reliable measure of the actual statistical properties of each tissue distribution, thus leading to an improper parameter selection. The low MMC values obtained for CJV at high noise levels seem to confirm this possibility (**Figure 4**). In line with previous studies (Chua et al., 2009), a moderate amount of spatial smoothing (i.e., 1 mm FWHM) led to a considerable increase of the CJV accuracy (**Figure 5**). The same solution did not prove to be as effective when using CV_WM_ and CV_GM_ instead of CJV.

After establishing that CJV in combination with spatial smoothing can yield a reliable estimation of INU correction parameters, we addressed the problem of how this metric could be effectively applied on actual MR data. Importantly, GM and WM masks are needed to measure the CJV, and different options exist as for deriving these masks from the actual MR images. One aspect to consider is that the INU correction algorithm of SPM is integrated with brain segmentation, such that GM and WM probability maps are automatically generated. This means that, in line of principle, it would be possible to estimate GM and WM maps for each input parameter configuration, and use them for the CJV calculation. Such a solution, however, does not permit an unbiased comparison across configurations, as the masks would be different case by case. Rather than using the SPM template masks registered to individual space, we implemented an approach that exploits the similarity between those template masks and the ones estimated from the SPM segmentation algorithm, which are subject-specific. By using the mean Dice Similarity Index (mDSI), we searched through the entire configurations space and selected a set of probability maps that had mDSI superior to a certain threshold.

For both WM and GM, all the maps satisfying the mDSI criteria were then averaged together, and then employed to create the actual masks. The latter ones were then used across all configurations for the CJV assessment. Our analysis on simulated data revealed that this approach can lead to the definition of masks that are very close to the ground truth masks and are much more precise than the SPM template masks registered to individual space (see **Figures 5A,B** and **7**). It is our opinion that the approach we implemented limits the possibility of deceptive CJV evaluations due to partial volume effects, which are typically present in the voxels including both WM and GM tissues.

To show the potential usefulness of the developed data-driven approach to estimate INU correction parameters, we used also actual MR images collected with 1.5 T, 3 T, and 7 T scanners, respectively. One of the main features that influences INU properties is indeed the strength of the static field (Boyes et al., 2008; Uwano et al., 2014). With increasing magnetic field, not only does the INU field magnitude rise, but also the INU spatial dynamic is more variable as a result of tissue-induced inhomogeneities (Mihara et al., 2005; Van De Moortele et al., 2005; Bernstein et al., 2006; Moser et al., 2012; Umutlu et al., 2014; Uwano et al., 2014). The CJV results for MR images at different magnetic fields suggested this metric to be sensitive to INU properties, since the minimum CJV value across the whole set of input parameters was different across MR images (**Figure 8**). For instance, a relatively low regularization parameter was identified as being more accurate for the 1.5 T image, consistent with a low frequency INU pattern compared to the underlying anatomical structures. The intensity had a consistent intensity drop at the center of the 3 T image. This might be related to a RF wavelength shortening as well as the coil sensitivity (Bernstein et al., 2006). Although this intensity inhomogeneity was still low frequency compared with anatomical brain structures, the CJV analysis suggested a larger level of regularization and the same FWHM level of the 1.5 T image. This was putatively due to a larger INU field magnitude. The 7 T image was characterized by a substantially different intensity inhomogeneity profile compared to the 1.5 T and 3 T images. In this case, the CJV values were more weighted toward higher regularization parameters that allowed the INU correction to better follow sharp inhomogeneity variations across the MR image.

When we extended our analysis on actual MR images to the whole KIRBY21 dataset, which was collected with a 3 T MR scanner, we could appreciate a very high stability of the configuration of input parameters selected by our data-driven method. This may indicate that the selected input configuration, rather than being subject-specific, more likely depends on the MR hardware and acquisition sequence used. It remains, however, to be verified if this finding for images collected at 3 T generalizes also to higher field strengths, for which tissue-induced inhomogeneities are more prominent. This may indeed lead to an increased inter-subject variability in the selected parameter configuration. Importantly, we also observed that the segmentation results for the same subject scanned in two separate sessions were more similar when using optimized than standard configurations (**Figure 9**). It is commonly accepted that intensity inhomogeneity primarily affects the accuracy of image segmentations (Belaroussi et al., 2006; Zheng et al., 2009). Accordingly, this finding might be taken as indirect evidence of an increased INU correction performance. Since we conducted the rest–retest analysis on a single dataset, we suggest that future studies are warranted to evaluate whether the increased INU correction performance is confirmed with other datasets, possibly collected with different scanners.

## Conclusion

To the best of our knowledge, this is the first study that addressed the problem of selecting the most appropriate input algorithm parameters for INU correction of structural MR images. Our analyses were based on the INU correction algorithm implemented in SPM, but the same approach can be in principle extended to any other INU correction algorithm requiring the selection of input parameters. In short, we conducted a comprehensive comparison of indirect metrics for the assessment of the INU correction results. We identified the CJV as the most accurate one, as long as the noise level in the INU-corrected image was controlled by means of spatial smoothing. Based on the CJV, we developed a data-driven approach aiding the selection of the parameters to be used for an accurate inhomogeneity correction in actual MR images. Our findings suggest that it is possible to tailor the parameter configuration of the INU correction algorithm based on the characteristics of the MR image to be processed, leading to a substantial improvement compared to the default parameter configuration. Since substantial progress is being made on the development of high-field MR scanners (Moser et al., 2012; Umutlu et al., 2014), the problem of INU correction is becoming increasingly important (Mihara et al., 2005; Van De Moortele et al., 2005; Bernstein et al., 2006; Uwano et al., 2014). The data-driven approach described here may contribute to address this problem by optimizing the performance of any given INU correction algorithm.

## Author Contributions

DM and NW designed the research. MG developed the method, analyzed the data and produced the results. DM and NW checked the correctness of the method and the results. MG and DM wrote a first draft of the manuscript, which was reviewed, and approved by all the authors.

## Conflict of Interest Statement

The authors declare that the research was conducted in the absence of any commercial or financial relationships that could be construed as a potential conflict of interest.

## Footnotes

1 http://www.fil.ion.ucl.ac.uk/spm

2 www.brainvoyager.com

3 brainweb.bic.mni.mcgill.ca/brainweb

4 http://biomedic.doc.ic.ac.uk/brain-development/index.php?n=Main.Datasets

5 http://www.nitrc.org/frs/shownotes.php?release_id = 2178

6 http://mri.kennedykrieger.org/databases.html
